# The dynamics of vaginal and rectal *Lactobacillus* spp. flora in subsequent trimesters of pregnancy in healthy Polish women, assessed using the Sanger sequencing method

**DOI:** 10.1186/s12884-018-1987-7

**Published:** 2018-08-29

**Authors:** Anna Dobrut, Tomasz Gosiewski, Wojciech Pabian, Malgorzata Bodaszewska-Lubas, Dorota Ochonska, Małgorzata Bulanda, Monika Brzychczy-Wloch

**Affiliations:** 1 Department of Molecular Medical Microbiology, Chair of Microbiology, Faculty of Medicine, Jagiellonian University Medical College, Czysta 18, 31-121, Krakow, Poland; 2 Clinical Department of Gynecological Endocrinology and Gynecology, Jagiellonian University Medical College, Kopernika 23, 31-501, Krakow, Poland; 3 Department of Immunology, Faculty of Biochemistry, Biophysics and Biotechnology of the Jagiellonian University, Gronostajowa 7, 30-387, Krakow, Poland

**Keywords:** *Lactobacillus* spp., Healthy pregnant women, Vaginal and rectal colonization

## Abstract

**Background:**

Lactobacilli play an important role in maintaining vaginal health and protection against bacterial infections in the genital tract. The aim of this study is to show the dynamics of changes of the vaginal and rectal *Lactobacillus* flora during pregnancy by using the Sanger sequencing method.

**Method:**

The study included 31 healthy pregnant women without clinical signs of genitourinary infections. The material was taken in the three trimesters of pregnancy by vaginal and rectal swabs and grown on the MRS agar quantitatively to estimate the number of *Lactobacillus* spp. [CFU/ml]. Afterwards, 3 to 8 morphologically different lactobacilli colonies were taken for identification. Bacterial species identification was performed by 16 s rDNA sequence fragment analyses using the Sanger method.

**Results:**

Among the patients tested, the most common species colonizing the vagina in the first trimester were: *L. crispatus* 29%, *L. gasseri* 19.4% and *L. rhamnosus* 16.1%, in the second trimester: *L. crispatus* 51.6%, *L. gasseri* 25.8%, *L. rhamnosus* 19.4% and *L. amylovorus* 16.1%, and in the third trimester the most common *Lactobacillus* species were: *L. crispatus* 25.8%, *L. gasseri* 25.8% and *L. johnsonii* 19.4%. In rectal species, the number decreased in the second and third trimesters in comparison to the first trimester (*p* = 0.003). An analysis of rectal dynamics showed that in the first trimester, the most common species were: *L. johnsonii* 19.4%, and *L. plantarum* 9.7%, in the second trimester: *L. crispatus* 9.7% and L. mucosae 6.5%, and in the third trimester: *L. casei* 9.7% and *L. rhamnosus* 9.7%. Individual dynamics of the *Lactobacillus* species composition showed variability, characterized by continuous, intermittent, or periodic colonization. The patients examined were mostly colonized by three *Lactobacillus* species in vagina (32.3%), whereas for the rectum, one *Lactobacillus* species during the whole pregnancy duration was common (32.3%).

**Conclusion:**

This study showed that in the examined group of healthy, pregnant Polish women, the vaginal *Lactobacillus* flora, both qualitative and quantitative, was stable during the three subsequent trimesters. In contrast, the number of rectal *Lactobacillus* species dramatically decreased after the first trimester.

## Background

The genus *Lactobacillus*, containing over 180 species, is composed of Gram-negative bacilli, which prefer microaerophilic or strictly anaerobic conditions. They form part of the human microbiome, as they colonize the lower part of the digestive tract, vagina and mouth. They are of particular importance in the female genital tract, where due to lactic acid synthesis, they reduce the pH, and therefore, protect it from bacterial or fungal infections. These bacteria also produce bacteriostatic and bactericidal substances, such as bacteriocins or hydrogen peroxide (H_2_O_2_), thereby eliminating the competing species in their environment [1, 2]. A reduction in the load of bacteria from the genus *Lactobacillus* with a simultaneous increase in the number of pathogenic anaerobic bacteria, such as *Gardnerella vaginalis*, *Prevotella* spp., or *Atopobium vaginalis*, is the main cause of bacterial vaginosis (BV) [3–5]. Maintaining the microbial balance of the vagina is of the greatest importance in the prevention of bacterial vaginosis; hence, in recent years, pharmaceuticals and dietary supplements containing probiotic strains of the genus *Lactobacillus* have been growing in popularity [6].

The microbial species composition of the vagina changes in individual periods of a woman’s life and depends on hormonal changes. In the course of fetal life, the reproductive system is devoid of microorganisms. During delivery, the newborn acquires the mother’s microbial flora and is under the influence of maternal estrogens in the first months of life. In infancy and early childhood, as a result of withdrawal from maternal hormones, bacteria of the genus *Lactobacillus* are completely absent, and the pH ranges from 6.0–8.0. They reappear when a woman enters puberty, together with the first menstruation. Shifts in the microbial flora of the vagina depend on hormonal changes. In the first stage of the menstrual cycle, when the body is under the control of estrogens, the number of bacteria from the genus *Lactobacillus* increases, while in the second phase of the cycle, as a result of progesterone dominance, the vaginal pH rises and the numbers of *Lactobacillus* fall. Changes in the vaginal flora also depend on other factors, such as: antibiotic therapy, hormone therapy, oral contraception or pregnancy [7].

During pregnancy, there are numerous hormonal changes in the woman’s body. As the pregnancy progresses, progesterone, prolactin and estrogen levels rise. There is also an increase in the concentration of thyroxine, a thyroid hormone, secreted. Additionally, the woman is under the influence of placental hormones, such as: placental gonadotropin, human placental lactogen, corticotropin, placental tyreotropin and steroid hormones [8]. Hormonal, as well as immunological and metabolic, changes in pregnancy significantly affect the microbiome composition [9–11].

Vaginal microbiota is dominated by bacteria from the genus *Lactobacillus* [12]. During pregnancy, significant modifications are also present in this niche. Bacterial diversity is reduced, while species stability and the number of species from the genus *Lactobacillus* are increased [12]. The predominant species among Asian and Caucasian pregnant women is *L. crispatus*, while *L. gasseri* is prevalent in Afro-Americans [13, 14].

Phenotypic methods used for species identification for the genus *Lactobacillus* are insufficient, therefore, molecular methods are currently most often employed. One such method is Sanger sequencing, which allows identification of species through analysis of the 16 s rDNA conservative sequence [15]. This technique is characterized by high sensitivity and specificity and makes it possible to obtain unambiguous and repeatable results [16].

Currently, there is a lack of data concerning the bacterial species composition as regards the genus *Lactobacillus* colonizing the vagina and rectum of pregnant women in Poland. Therefore, the objective of the present study is to analyze the dynamics of vaginal and rectal microbiota composition, formed by the species from the genus *Lactobacillus* in the three subsequent trimesters of pregnancy, which were identified using the Sanger molecular sequencing method. In these studies, we first determine the species composition of the *Lactobacillus* genus in the vagina and rectum, and then the examined effect of the trimesters of pregnancy on changes in the dynamics of colonization by lactobacilli. Research conducted on a healthy pregnant population may be a starting point for studies in groups of women with a specific underlying disease, e.g. diabetes.

## Methods

### Specimen collection and bacterial strains

Initially, the study included 42 healthy pregnant women aged 22–35, who were subjected to microbiological testing for the presence of bacteria from the genus *Lactobacillus* in the three subsequent trimesters of pregnancy. However, due to the prolonged research period, 11 women did not maintain the continuity of their doctors’ visits. Consequently, the analysis comprised 31 patients, who underwent controlled testing in each trimester. The consent obtained from the participants was both informed and written. The inclusion and exclusion methodology has been described in our previous paper [17]. Inclusion criteria: women between the ages of 18 and 40; pregnant women in the first trimester of gestation (1st-13th week of gestation); without clinical symptoms of genitourinary infections requiring antibiotherapy; confirmed physiological composition of genitourinary tract flora according to the 10-point Nugent score (results: a value between 0 and 6); written permission to take part in the study. Exclusion criteria: women under 18 and over 40; pregnant women with the so-called high-risk pregnancy; rupture of membranes; gestational diabetes; antibiotherapy within a period of up to 30 days before getting pregnant or during gestation; diagnosis of bacterial vaginosis on the basis of a direct smear from a vaginal swab stained by Gram’s method (results: 7–10 points in the 10-point Nugent score) and on the basis of culture; clinical symptoms of genitourinary infection requiring antibiotherapy; lack of written permission to take part in the study. The tests have been approved by the Bioethics Committee (No. KBET/47/B/2009.), and the patients tested gave their written consent to participate in the study.

The material was taken from the vagina and rectum using sterile swabs during routine visits to the gynecologist. The vaginal specimen was taken using two swabs moistened with 0.9% NaCl, and the rectal one (past the anal sphincter) with the use of one swab moistened with 0.9% NaCl. Additionally, vaginal pH was measured using test strips (Sigma-Aldrich). The material taken from the vagina using the first swab made it possible to prepare a specimen, which was later stained with the Gram method in order to assess the vaginal flora according to the Nugent score of 1–10 [18].

The second swab with a vaginal smear was placed in 1 ml 0.9% NaCl, vortexed thoroughly, and then subjected to a series of dilutions by transferring 100 μl of the sample each time to next tubes with 0.9 ml 0.9% NaCl. The resulting dilutions were obtained 10^0^–10^− 7^, from which, each time, 100 μl was subjected to culture on a solid MRS medium (Difco) in order to estimate the number of bacteria [CFU/ml]. Culturing was carried out under anaerobic conditions at 37 °C in the presence of a generator for culturing anaerobic bacteria, GENbox anaer (bioMérieux), for 72 h. When the culture was finished, all of the various colonies were screened on the MRS medium and incubated for 24–48 h. Next, for the individual isolates, the preparations were made and stained by the Gram method. The morphology and morphologies and the method of staining were evaluated under a microscope (Olympus) at 100× magnification. Additionally, during the isolation, for every isolate (colony morphotype), the number of colonies with the same morphotype was counted, which then, considering dilution, was converted to CFU/ ml for the individual isolates.

An analogous procedure for culture and isolation for bacteria of the genus *Lactobacillus* was carried out for swabs taken from the patients’ rectums.

The isolated colonies with the *Lactobacillus* morphotype were studied using phenotypic methods including culturing under anaerobic conditions, assessment of the ability to produce H_2_O_2_ using the Peroxide Test (Merck), vancomycin resistance testing [VA, 5 μg] (Oxoid) with the use of the disk diffusion method according to the Bergey’s manual [19]. Affiliation to the genus was assessed on the basis of API 20A (bioMérieux) and API 50CH (bioMérieux) test result analyses. Bacterial strains were stored in the ViabankTM system (VWR) at − 70 °C.

### DNA isolation

In order to revive bacteria, and then isolate DNA for sequencing, they were grown for 48 h at 37 °C on the MRS (Difco) solid medium under anaerobic conditions in the presence of the GENbox anaer (bioMérieux) generator for anaerobic bacteria cultivation.

Then, one colony was collected and cultured in 3 ml of the MRS liquid medium at 37 °C for 24 h under anaerobic conditions. The isolation of bacterial DNA was carried out using the commercial GeneMATRIX Bacterial & Yeast Genomic DNA Purification Kit (EURx) according to the procedure developed by the manufacturer with the addition of 3.5 μl mutanolysin (Sigma-Aldrich) and 7 μl lysozyme (Sigma-Aldrich). The concentration and purity of the isolated DNA was assessed using NanoDrop (Thermo Scientific).

### Polymerase chain reaction (PCR)

Amplification of bacterial DNA was done using a universal pair of primers TP16U1 (AGAGTTTGATCMTGGCTCAG) and RT16U6 (ATTGTAGCACGTGTGTNGCCC) (Genomed) at concentration 0.3 μM [16]. The reaction mixture also comprised 25 μl of 2xPCR Master Mix RAPID (A&A Biotechnology) commercial mixture, 17 μl of RNase-free water (A&A Biotechnology) and 2 μl of bacterial DNA. The PCR was carried out using the T100 Thermal Cycler (BioRad) according to the program: preliminary denaturation at 94 °C for 2 min prior to 25 repeated cycles: 1 min denaturation at 92 °C, 1 min introduction of primers at 55 °C and 1.5 min elongation at 72 °C finished with 5-min final extension [16]. When the PCR was complete, the product of amplification was subjected to electrophoretic separation on 1.5% agarose gel (Prona ABO) in 1× TBE buffer (Sigma-Aldrich) with the addition of ethidium bromide (Sigma-Aldrich) for 60 min at voltage 5 V/cm. The results of the electrophoretic separation were documented with the use of the Gel Doc 2000 (BioRad) system in the presence of UV light.

### Sanger sequencing and result analysis

The products of amplification measuring 1233 bp were subjected to Sanger sequencing (Genomed). Reference strains used as models represented the following species: *L. acidophilus* ATCC 4356, *L. fermentum* ATCC 20052, *L. plantarum* ATCC 20174, *L. plantarum* ATCC 14431, *L. delbrueckii ssp. bulgaricus* ATCC 20074, *L. crispatus* ATCC 20225 and *L. gasseri* ATCC 20243. Then, using version 1.7 ChromasPro (Technelysium Pty Ltd) software, consensus sequences were generated, which were entered into the NCBI database (National Center of Biotechnology Information Database with BLAST software) (http://www.ncbi.nlm.nih.gov/blast), in order to identify the bacterial species. The result of the analysis was considered positive if the similarity of the sequence entered into the database was ≥98% compared with the model sequences in the BLAST database.

### Statistical analysis

The statistical analysis was carried out on the basis of descriptive statistics, the Friedman test and the Fisher tests were used to determine whether the presence of a particular species was changing bacteria in subsequent trimesters, and the Wilcoxon test was used to determine if the number of species colonizing a given niche changes in subsequent trimesters. Statistical analysis was carried out by using software from the IBM SPSS Statistics 23 package. The value of *p* > 0.05 was considered significant.

There was also a detailed analysis of the dynamics of species composition of bacteria from the genus *Lactobacillus* carried out for 31 patients with a full set of tests, understood as swabs taken in each trimester of the pregnancy. The testing encompassed both the qualitative and quantitative composition of individual *Lactobacillus* species, for both the vagina and rectum.

## Results

**Fig. 1:**
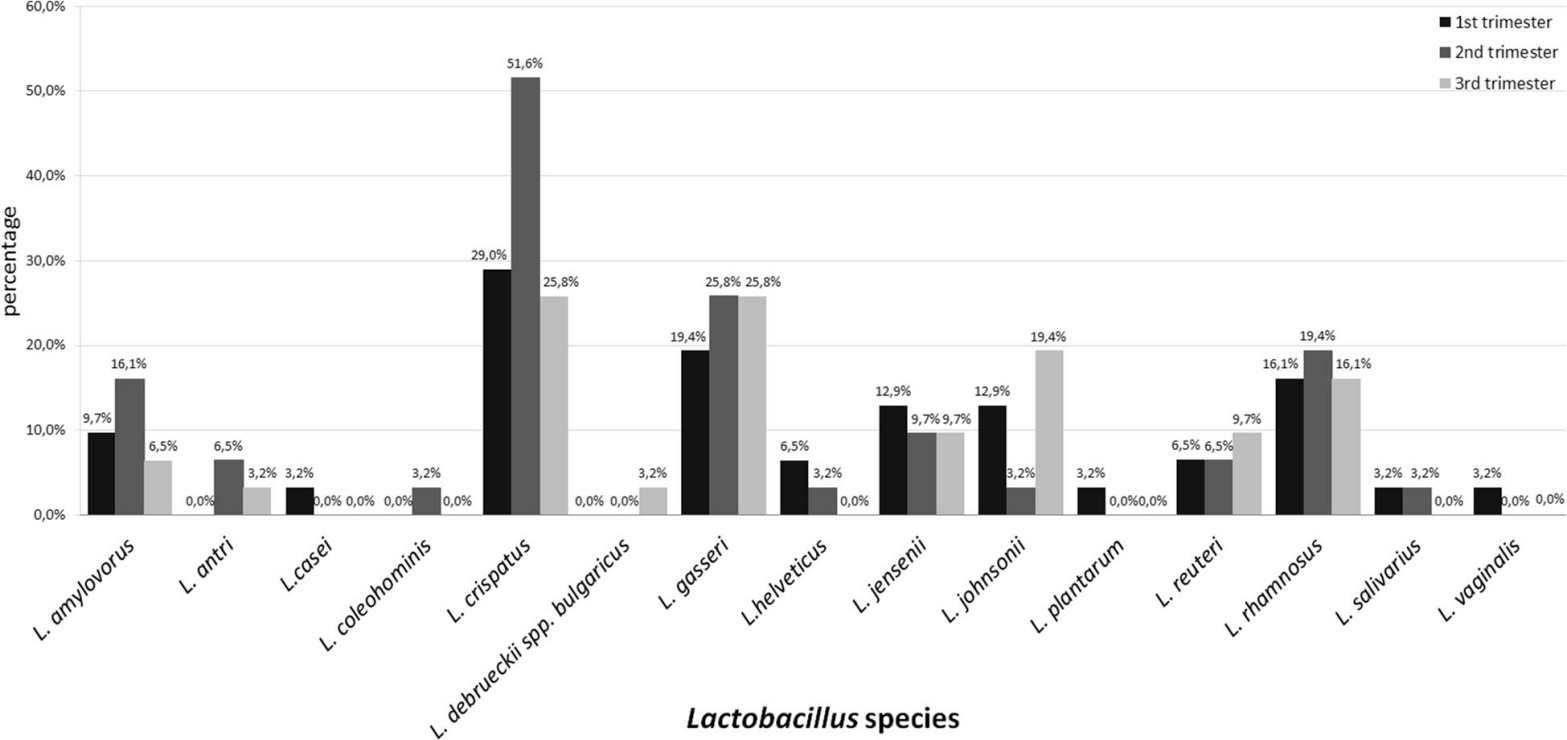
The percentage of particular *Lactobacillus* species colonizing the vagina in subsequent trimesters of pregnancy

The employment of Sanger sequencing allowed identification of 15 different species of *Lactobacillus* which colonize the vagina at least once during pregnancy in tested population. In the first trimester, 12 species were determined, in the second trimester: 11, and in the third: 9. A qualitative analysis of the subsequent trimesters demonstrated that, in the first trimester, the vaginal species most commonly isolated were: *L. crispatus* 29% (*n* = 9), followed by *L. gasseri* 19.4% (*n* = 6), *L. rhamnosus* 16.1% (*n* = 5), *L. jensenii* 12.9% (*n* = 4), *L. johnsonii* 12.9% (*n* = 4), *L. amylovorus* 9.7% (*n* = 3), *L. helveticus* 6.5% (*n* = 2), *L. reuteri* 6.5% (*n* = 2), *L. casei* 3.2% (*n* = 1), *L. plantarum* 3.2% (*n* = 1), *L. salivarius* 3.2% (*n* = 1) and *L. vaginalis* 3.2% (*n* = 1). On the other hand, in the second trimester, the most often present species were as follows: *L. crispatus* 51.6% (*n* = 16), *L. gasseri* 25.8% (*n* = 8), *L. rhamnosus* 19.4% (*n* = 6), *L. amylovorus* 16.1% (*n* = 5), *L. jensenii* 9.7% (*n* = 3), *L. antri* 6.5% (*n* = 2), *L. reuteri* 6.5% (*n* = 2), *L. coleohominis* 3.2% (*n* = 1), *L. helveticus* 3.2% (*n* = 1), *L. johnsonii* 3.2% (*n* = 1) and *L. salivarius* 3.2% (*n* = 1). While in the third trimester, the most frequent species were: *L. crispatus* 25.8% (*n* = 8), *L. gasseri* 25.8% (*n* = 8), followed by *L. johnsonii* 19.4% (*n* = 6), *L. rhamnosus* 16.1% (*n* = 5), *L. jensenii* 9.7% (*n* = 3), *L. reuteri* 9.7% (*n* = 3), *L. amylovorus* 6.5% (*n* = 2), *L. antri* 3.2% (*n* = 1) and *L. delbrueckii subsp. bulgaricus* 3.2% (*n* = 1) (Fig. 1).

**Fig. 2:**
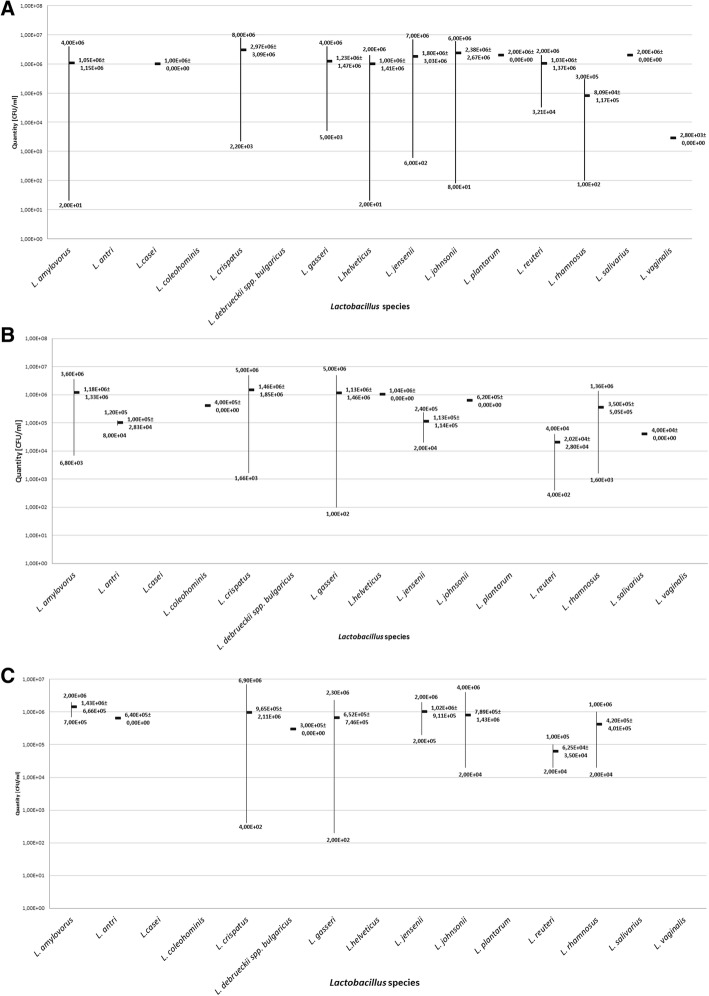
The numbers [CFU/ml] of particular *Lactobacillus* species colonizing the vagina in subsequent trimesters of pregnancy. Legend: **a** the number of particular species from the genus *Lactobacillus* colonizing the vagina in the first trimester of gestation, **b** the number of particular species from the genus *Lactobacillus* colonizing the vagina in the second trimester of gestation, **c** the number of particular species from the genus *Lactobacillus* colonizing the vagina in the second trimester of gestation

Analysis of the numbers of *Lactobacillus* [CFU/ml] colonizing the vagina in the three subsequent trimesters of pregnancy demonstrated that the most bacteria could be found in the first trimester, 1.10E + 06 CFU/ml, on average, while in the ensuing trimesters the average number was 4.30E + 05 CFU/ml in the second one and 4.19E + 05 CFU/ml in the third. Detailed results are presented in Table 1, Fig. 2a, b and c.

**Table 1:** Average bacterial number [CFU/ml] and standard deviation of particular Lactobacillus species colonizing vagina and rectum

*Lactobacillus* species			1st trimester		2nd trimesterr		3rd trimeste	
			Average bacterial number [CFU/ml]	±SD [CFU/ml]	Average bacterial number [CFU/ml]	±SD [CFU/ml]	Average bacterial number [CFU/ml]	±SD [CFU/ml]
vagina	1	*L. amylovorus*	1,05E + 06	1,15E + 06	1,18E + 06	1,33E + 06	1,43E + 06	6,66E + 05
	2	*L. antri*	0,00E + 00	0,00E + 00	1,00E + 05	2,83E + 04	6,40E + 05	0,00E + 00
	3	*L. casei*	1,00E + 06	0,00E + 00	0,00E + 00	0,00E + 00	0,00E + 00	0,00E + 00
	4	*L. coleohominis*	0,00E + 00	0,00E + 00	4,00E + 05	0,00E + 00	0,00E + 00	0,00E + 00
	5	*L. crispatus*	2,97E + 06	3,09E + 06	1,46E + 06	1,85E + 06	9,65E + 05	2,11E + 06
	6	*L. debrueckii subsp. bulgaricus*	0,00E + 00	0,00E + 00	0,00E + 00	0,00E + 00	3,00E + 05	0,00E + 00
	7	*L. gasseri*	1,23E + 06	1,47E + 06	1,13E + 06	1,46E + 06	6,52E + 05	7,46E + 05
	8	*L. helveticus*	1,00E + 06	1,41E + 06	1,04E + 06	0,00E + 00	0,00E + 00	0,00E + 00
	9	*L. jensenii*	1,80E + 06	3,03E + 06	1,13E + 05	1,14E + 05	1,02E + 06	9,11E + 05
	10	*L. johnsonii*	2,38E + 06	2,67E + 06	6,20E + 05	0,00E + 00	7,89E + 05	1,43E + 06
	11	*L. plantarum*	2,00E + 06	0,00E + 00	0,00E + 00	0,00E + 00	0,00E + 00	0,00E + 00
	12	*L. rhamnosus*	1,03E + 06	1,37E + 06	2,02E + 04	2,80E + 04	6,25E + 04	3,50E + 04
	13	*L. reuteri*	8,09E + 04	1,17E + 05	3,50E + 05	5,05E + 05	4,20E + 05	4,01E + 05
	14	*L. salivarius*	2,00E + 06	0,00E + 00	4,00E + 04	0,00E + 00	0,00E + 00	0,00E + 00
	15	*L. vaginalis*	2,80E + 03	0,00E + 00	0,00E + 00	0,00E + 00	0,00E + 00	0,00E + 00
rectum	1	*L. acidophilus*	2,00E + 02	0,00E + 00	0,00E + 00	0,00E + 00	0,00E + 00	0,00E + 00
	2	*L. amylovorus*	2,00E + 02	0,00E + 00	0,00E + 00	0,00E + 00	0,00E + 00	0,00E + 00
	3	*L. casei*	1,08E + 04	1,30E + 04	1,20E + 03	0,00E + 00	2,20E + 05	3,64E + 05
	4	*L. crispatus*	1,25E + 06	1,76E + 06	7,68E + 04	1,07E + 05	3,05E + 05	4,31E + 05
	5	*L. fermentum*	0,00E + 00	0,00E + 00	0,00E + 00	0,00E + 00	0,00E + 00	0,00E + 00
	6	*L. frumenti*	1,40E + 03	0,00E + 00	0,00E + 00	0,00E + 00	0,00E + 00	0,00E + 00
	7	*L.gasseri*	6,20E + 03	0,00E + 00	1,60E + 05	0,00E + 00	0,00E + 00	0,00E + 00
	8	*L. jensenii*	5,50E + 03	6,36E + 03	0,00E + 00	0,00E + 00	8,00E + 01	0,00E + 00
	9	*L. johnsonii*	9,17E + 04	1,14E + 05	2,00E + 02	0,00E + 00	0,00E + 00	0,00E + 00
	10	*L. mucoase*	6,00E + 02	0,00E + 00	1,01E + 04	1,40E + 04	0,00E + 00	0,00E + 00
	11	*L. paracasei*	2,00E + 05	0,00E + 00	0,00E + 00	0,00E + 00	0,00E + 00	0,00E + 00
	12	*L.plantarum*	1,40E + 04	2,25E + 04	6,20E + 03	0,00E + 00	0,00E + 00	0,00E + 00
	13	*L. reuteri*	0,00E + 00	0,00E + 00	2,00E + 02	0,00E + 00	0,00E + 00	0,00E + 00
	14	*L. rhamnosus*	6,00E + 04	0,00E + 00	0,00E + 00	0,00E + 00	7,18E + 05	1,23E + 06
	15	*L. ruminis*	2,00E + 03	8,49E + 02	0,00E + 00	0,00E + 00	2,00E + 04	0,00E + 00
	16	*L. sakei*	0,00E + 00	0,00E + 00	0,00E + 00	0,00E + 00	1,00E + 02	0,00E + 00
	17	*L. salivarius*	1,50E + 05	2,12E + 05	6,00E + 02	0,00E + 00	1,20E + 02	0,00E + 00

In the group of women studied, during the entire pregnancy period, the vagina was colonized by 5 species from the genus *Lactobacillus* in 9.7% (*n* = 3) of the patients; in 3.2% (*n*- = 1) of patients there were 4 species present, and most often, because in as many as 32.3% (*n* = 10) of patients, the presence of 3 various species was recorded; in 29% (*n* = 9) of patients there were two species, while in 16.1% of women (*n* = 5) there was one species present. In 9.7% (*n* = 3) of the patients, throughout their pregnancies, there were no *Lactobacillus* species determined.

Analysis of the dynamics of the percentage share of the *Lactobacillus* species in the microbiota composition showed differences in the general and individual colonization of the genital tract in pregnant women. In the group of patients subjected to the study, 58% were colonized in each trimester with at least one *Lactobacillus* species, in 19%, *Lactobacillus* was present in two successive trimesters. In 6.5% of women, *Lactobacillus* colonized the vagina in the first trimester. Similarly, in 6.5% of women, *Lactobacillus* was present in only one trimester. However, these differences were not statistically significant, which may stem from the fact that the studied group of patients was too small.

Analysis of the frequency of occurrence of particular species colonizing the vagina in individual patients showed diversity. In 9.7% of the patients, *L. crispatus* was present throughout the pregnancy, in 22.6% of women this species was identified in two subsequent trimesters, while in 6.5% of the cases, *L. crispatus* appeared in the first and third trimesters. *L. gasseri*, in the case of one patient (3.2%), was present in the vagina throughout the entire pregnancy, three patients (9.7%) were colonized in two successive trimesters, and two others (6.5%) in the first and third trimesters. In two women (6.5%), *L. rhamnosus* colonized the vagina in the three trimesters of gestation, while two others (6.5%) were colonized by this species in two subsequent trimesters. In three women (9.7%), *L. jensenii* was present in two subsequent trimesters, while *L. johnsonii* was identified in the first and third trimesters in two patients (6.5%). *L. reuteri* colonized the vagina for two subsequent trimesters in two patients (6.5%), and in one (3.2%), it appeared in the first and the last trimesters*. L. amylovorus* and *L. antri* were isolated in two consecutive trimesters in 3.2% of the studied patients. *L. casei*, *L. delbrueckii sb.bulgaricus*, *L. helveticus*, *L. salivarius*, *L. vaginalis*, *L. coleohominis* and *L. plantarum* were found only in one trimester.

In the rectal isolate pool studied, 17 different species from the genus *Lactobacillus* that appeared at least once throughout the entire period of pregnancy were identified. The most, as many as 14, species colonized the rectum in the first trimester; in consecutive trimesters, this number decreased by around 50%: in the second trimester, 8 species were identified, and in the third, there were only 7 (*p* = 0.003).

**Fig. 3:**
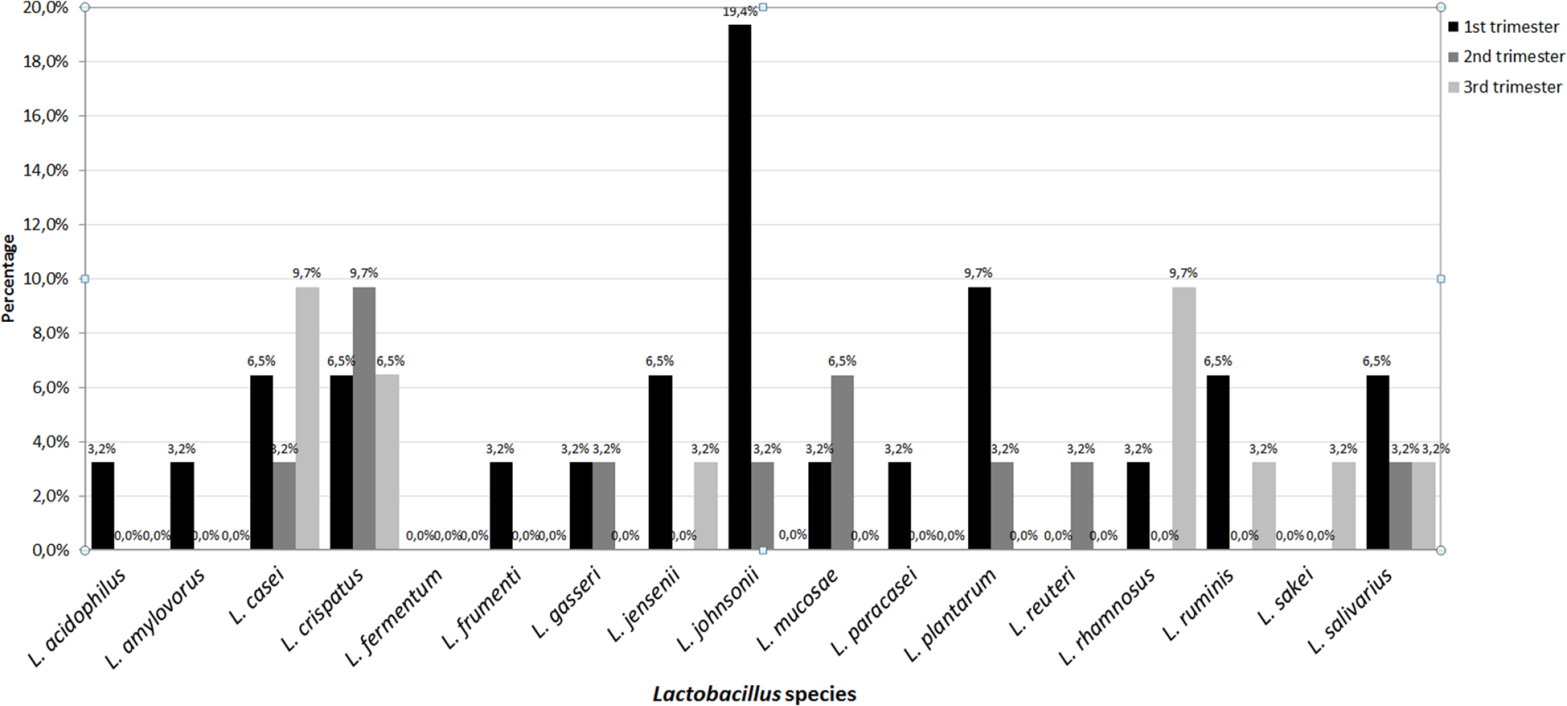
The percentage of particular *Lactobacillus* species colonizing the rectum in subsequent trimesters of pregnancy

**Fig. 4:**
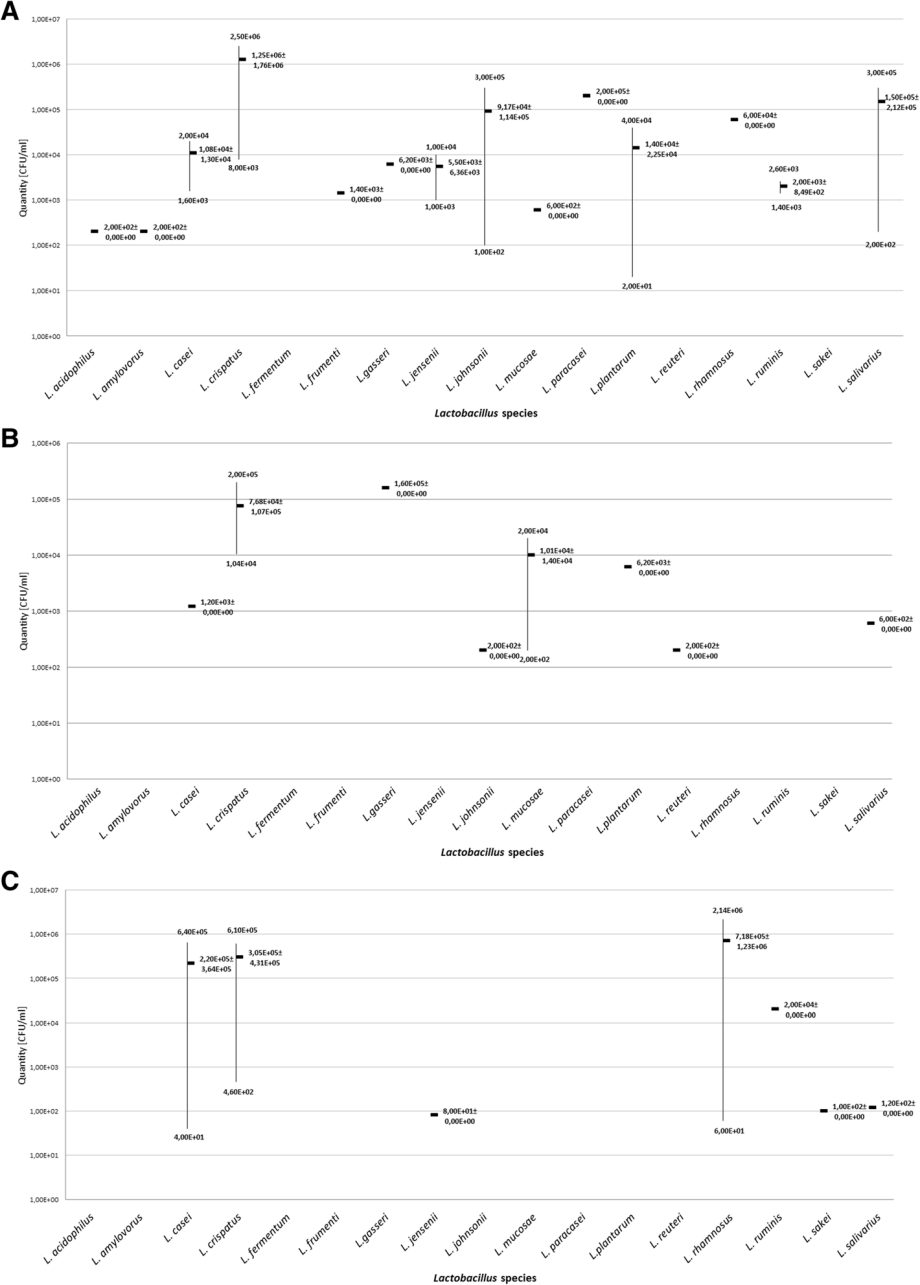
The numbers [CFU/ml] of particular *Lactobacillus* species colonizing the rectum in subsequent trimesters of pregnancy. Legend: **a** the number of particular species from the genus *Lactobacillus* colonizing the rectum in the first trimester of gestation, **b** the number of particular species from the genus *Lactobacillus* colonizing the rectum in the second trimester of gestation, **c** the number of particular species from the genus *Lactobacillus* colonizing the rectum in the second trimester of gestation

Analysis of the dynamics of the species composition in the rectum in the three subsequent trimesters of pregnancy demonstrated that, in the first trimester, the most frequently encountered species were the following: *L. johnsonii* 19.4% (*n* = 6), *L. plantarum* 9.7% (*n* = 3), *L. casei* 6.5% (*n* = 2), *L. crispatus* 6.5% (*n* = 2), *L. jensenii* 6.5% (*n* = 2), *L. ruminis* 6.5% (*n* = 2), *L. salivarius* 6.5% (*n* = 2), *L. acidophilus* 3.2% (*n* = 1), *L. amylovorus* 3.2% (*n* = 1), *L. frumenti* 3.2% (*n* = 1), *L. gasseri* 3.2% (*n* = 1), *L. mucosae* 3.2% (*n* = 1), *L. paracasei* 3.2% (*n* = 1) and *L. rhamnosus* 3.2% (*n* = 1). In the second trimester, the most frequent species was *L. crispatus* 9.7% (*n* = 3), followed by *L. mucosae* 6.5% (*n* = 2), *L. casei* 3.2% (*n* = 1), *L. gasseri* 3.2% (*n* = 1), *L. johnsonii* 3.2% (*n* = 1), *L. plantarum* 9.1% (*n* = 1), *L. reuteri* 9.1% (*n* = 1), *L. salivarius* 9.1% (*n* = 1). On the other hand, in the third trimester, the species isolated most often were: *L. casei* 9.7% (*n* = 3), *L. rhamnosus* 9.7% (*n* = 3) and *L. crispatus* 6.5% (*n* = 2) and then *L. jensenii* 3.2% (*n* = 1), *L. ruminis* 3.2% (*n* = 1), *L. sakei* 3.2% (*n* = 1), *L. salivarius* 3.2% (*n* = 1) (Fig. 3). Statistically significant differences as regards the incidence was shown for the *L. johnsonii* (*p* = 0.014) species. Analysis of the numbers of [CFU/ml] Lactobacillus in the three subsequent trimesters, similarly as in the case of the vagina, showed a slight decrease in the numbers in successive trimesters. In the first trimester, the average numbers of the *Lactobacillus* bacteria was 1.06E + 05 CFU/ml, while in the second trimester it was 1.50E + 04 CFU/ml, and in the third: 7.43E + 04 CFU/ml. Detailed results are shown in Table 1, Fig. 4a, b and c. The analysis of the numbers of individual species demonstrated significant differences in the case of *L. johnsonii* (*p* = 0.003).

In the group of women studied, the most species of *Lactobacillus*, because it was as many as five different ones, were isolated from only one patient (3.2%); in three patients (9.7%), three different *Lactobacillus* species were isolated, and in nine (29%) women, two various species were isolated, while in ten (32.3%) women, one species was present throughout their pregnancies. In eight patients (25.8%), there was no determination of bacteria from this species in the rectum throughout the gestation.

The studied pool of all *Lactobacillus* strains, isolated from both the vagina and anus, were also described in terms of their ability to produce H_2_O_2_. It was demonstrated that 84% of all the isolates examined were H2O2-positive and 16% were H2O2-negative. Among the H2O2-producing species, there were: *L. acidophilus*, *L. casei*, *L. crispatus*, *L. delbrueckii subsp. bulgaricus*, *L. gasseri*, *L. helveticus*, *L. jensenii*, *L. johnsonii* and *L. reuteri*.

## Discussion

The Sanger method has found broad application in species identification as regards the *Lactobacillus* strains, both in science [16, 20], and the food industry, particularly in typing species responsible for fermentation of dairy products [21].

Based on this method, this study has identified 15 various species of *Lactobacillus* colonizing the female reproductive tract. The same outcome was obtained by Anderson et al., who also identified 15 *Lactobacillus* species colonizing the vagina in the group of German patients studied [16]. Despite the fact that the qualitative and quantitative composition of bacteria from the genus *Lactobacillus* depends on numerous factors, as it is clear from literature data, the species that most often colonize the reproductive tract in healthy women are *L. crispatus*, *L. jensenii* and *L. gasseri* [22]. These observations coincide with the results of our studies, which demonstrate that the dominant species was *L. crispatus*, while *L. gasseri* was the second most commonly isolated species colonizing the vagina in each of the three trimesters of gestation. Our results correspond with studies conducted on Polish women by Martirosian et al. [23], who compared the vaginal flora of healthy women and patients after a kidney transplantation. She identified two species in healthy women, which were *L. crispatus* and *L. gasseri*, however two species remained unidentified. This might be caused by different methodology that was based on the PCR reaction. Comparable results were also described by Pendharkar et al., who examined the microbiome in women living in South Africa. They discovered the predominance of *L. crispatus*, *L. iners*, *L. jensenii*, *L. gasseri* and *L. vaginalis* [5]. On the other hand, investigations carried out by Alioua et al., aiming at comparing the microbiological flora of the reproductive tract in healthy women and those with a diagnosis of vaginosis, found the presence of the following species: *L. crispatus*, *L. jensenii*, *L. johnsonii*, *L. acidophilus*, *L. iners* and *L.delbrueckii* in the female vagina which, according to the Nugent score, were classified as being below 2.66 [24]. Examinations conducted by Korean scientists, using qPCR, also reflected the results of analyses presented in this study. They showed that, in the group of patients studied, the species isolated most often were *L. crispatus*, *L. gasseri*, *L. jensenii*, *L. vaginalis* and *L. iners* [25].

Analysis of the species dynamics for individual patients demonstrated that 32.3% of women were colonized with three various species of *Lactobacillus* for the entire pregnancy, and a similar finding was obtained by Petricevic et al. [14], who demonstrated the presence of three species in 39% of their pregnant patients. Likewise, in the case of colonization with four species, in which Petricevic found their presence in 4% of the patients, our testing gave a result of 3.2%.

Characterization of the species composition in the rectum found a greater number of species in comparison with the vaginal flora. In this examination, 17 various *Lactobacillus* species were identified, however, their diversity in individual trimesters of gestation was smaller than in the case of the vagina. In the patient group studied, 16.1% were colonized in the rectum with bacteria from the genus *Lactobacillus* throughout their pregnancies, however, the species composition was subject to significant changes (*p* = 0.003), as it was demonstrated in the analysis, none of the patients was colonized by the same species in the three consecutive trimesters. The majority of the species appeared only once during the entire pregnancy. The exceptions were species such as *L. crispatus*, which in the case of two patients (6.5%), was present in two subsequent trimesters, and in one (3.2%), in the first and last trimester. In 3.2% of the patients, *L. mucosae* was isolated in the first and second trimesters of gestation, while *L. ruminis* was found in the first and third trimesters. We presume that the decrease in the microorganisms in the rectal sample during three trimesters might be caused by physiological changes in woman’s body, especially in the third trimester, such as bloating and hemorrhoids, which can affect the imbalance in this niche, as well as impede proper material collection. We believe this should be verified in the extended patient group.

The analysis of the species composition of *Lactobacillus* colonizing the rectum in pregnant women showed that, out of the 17 identified species, the predominant ones were *L. crispatus*, *L. casei* and *L. johnsonii*. The finding obtained is confirmed by the work by Song et al., who using the multiplex PCR reaction, identified the following species in feces: *L. acidophilus*, *L. crispatus*, *L. fermentum*, *L. gasseri*, *L. jensenii*, *L. plantarum*, *L. reuteri*, *L. rhamnosus*, *L. salivarius*, *L. paracasei* and *L. delbrueckii* [26]. While Cooperstock et al., when studying the feces of Europeans, both neonates and adults, demonstrated the presence of species such as *L. salivarius*, *L. fermentum* and *Lactobacillus* species belonging to the acidophilus group (among others, *L. crispatus*, *L. gasseri*, *L. johnsonii*, *L. amylovorus*) [27]. Similar findings were presented by a Swedish scientific team, according to which, the species most frequently isolated from the anus were: *L. plantarum*, *L. rhamnosus* and *L. paracasei* [28].

In our paper, we had characterized the dynamics of the *Lactobacillus* species in three subsequent trimesters in 32 healthy pregnant Polish women. We believe that due to the lack of data from our geographical region, the strengths of our study lie in testing this particular group of patients, the long testing duration of individual patients, and the applied methodology, which included the quantitative cultivation of the collected material and species identification by the Sanger sequencing method. Nevertheless, some limitation of our work can be observed in the lack of a correspondingly large group of patients, which would make it possible to determine more statistically significant differences. To improve the statistical aspect of our project, an extension of the study group could constitute a starting point for further investigation, which compares *Lactobacillus* flora in healthy women in physiological pregnancy to patients with a pathology of pregnancy, such as gestational diabetes, endocrine and immunological disorders.

## Conclusion

In the present study, we have demonstrated that the microbiota consisting of the *Lactobacillus* species in the vagina in pregnant women is stable, both quantitatively and qualitatively, in the three subsequent trimesters of gestation. While in the rectum, the numbers of *Lactobacillus* species in the first trimester is significantly higher. Additionally, we have shown that the dominant vaginal species is *L. crispatus*, while the prevalent rectal species is *L. johnsonii*. The species composition was subject to change in the case of both individual patients, as well as in the whole group subjected to the study; however, these differences were not statistically significant.
